# Education Research: ANSWER: A Multimodal Teaching Intervention for Neurology Undergraduate Medical Education: A Randomized Crossover Control Trial

**DOI:** 10.1212/NE9.0000000000200238

**Published:** 2025-09-08

**Authors:** Liah McElligott, Muirne Spooner, Caitriona Cahir, Diane Gillan, Hijaz Adenan, Grainne Mulkerrin, Susan Byrne, Norman Delanty, Claire Hevican, Arnold Hill, Noel Gerry McElvaney, Eavan McGovern

**Affiliations:** 1 School of Postgraduate Studies, Royal College of Surgeons in Ireland, Dublin;; 2 School of Medicine, Royal College of Surgeons in Ireland, Dublin;; 3 Data Science Centre, School of Population Health, Royal College of Surgeons in Ireland, Dublin;; 4 Department of Psychology, Royal College of Surgeons in Ireland and Beaumont Hospital, Dublin;; 5 Department of Neurology, Beaumont Hospital, Dublin;; 6 Department of Paediatrics, Children's Health Ireland, Dublin;; 7 Future Neuro SFI, Research Centre for Neurological Disease, Royal College of Surgeons in Ireland, Dublin;; 8 Department of Surgery, Royal College of Surgeons in Ireland, Dublin.

## Abstract

**Background and Objectives:**

Multimodal education uses cognitive learning theory strategies. Neurophobia, “the fear of neurology and clinical neuroscience,” affects medical students and doctors worldwide. Novel approaches to neurology undergraduate education can improve undergraduate knowledge, enhance student perception of clinical neurology, and reduce neurophobia. We examine the effect of *ANSWER* (*Analogy, Switch to Clinical, Embody the Signs and Recall Learning*), a multimodal undergraduate neurology teaching intervention, on neurophobia and neurology knowledge in final-year medical students.

**Methods:**

Final-year medical students were randomly distributed into 2 groups: an intervention group (*ANSWER* teaching) and a control group (usual teaching). A randomized crossover design was used. Knowledge acquisition was assessed using the multiple-choice question examination (*MCQE*). Neurophobia was assessed using a validated scale, *Neuro-Combined Measure* (*NCM*). The Kirkpatrick model evaluated the teaching program.

**Results:**

Seventy-seven final-year medical students participated. Neurology knowledge significantly improved after the intervention (MCQE: median = 14, interquartile range (IQR) = 4 vs 11, IQR = 3; *p* < 0.001; *r*_ββ_ = 0.77), and neurophobia significantly decreased (NCM: median = 26, IQR = 7 vs 29, IQR = 7; *p* = 0.004; *r*_ββ_ = 0.51). A four-week washout demonstrated sustained improvements (MCQE: *p* < 0.001, *r*_ββ_ = 1.00; NCM: *p* < 0.001, *r*_ββ_ = 1.00). The control group showed no significant change in knowledge (*z* = −1.78, *p* = 0.075, *r*_ββ_ = 0.30) or neurophobia (*z* = 1.10, *p* = 0.27, *r*_ββ_ = 0.21). Across groups, MCQE median scores increased, with the most significant gain observed in the intervention group (11 [95% CI 9.80–12.20] to 14 [95% CI 12.57–15.43]). NCM scores declined in both the intervention and washout groups but remained stable in the control group. Most participants reported that the intervention improved their neurology knowledge and clinical performance.

**Discussion:**

*ANSWER* teaching demonstrated improvement in neurology knowledge and neurophobia among final-year medical students. Students reported that *ANSWER* teaching improved their understanding of neurology and preparedness for examinations, suggesting that it is a promising tool for teaching neurology.

## Introduction

Neurologic disorders are a leading cause of disease burden and mortality worldwide,^1^ and neurology remains under-resourced in health care settings.^2-4^ Given the prevalence and impact of neurologic conditions, all doctors, regardless of specialty, should possess fundamental neurology knowledge and skills to ensure accurate diagnosis, timely referral, and effective patient management. There is a shortage of neurologists globally,^2,5,6^ which further intensifies the burden on nonspecialist health care providers to manage neurologic cases.

Health care professionals are first introduced to neuroscience and clinical neurology during medical school.^7-9^ Medical students consistently rank neuroscience subjects such as neuroanatomy difficult.^10-13^ Clinical years compound this difficulty when medical students attempt to integrate neuroscience subjects with the clinical encounter.^14^ These challenges can lead to neurophobia, *fear of neurology and clinical neuroscience*^15^; poor examination performance;^14,16^ and low match rates for neurology residencies.^10,17-20^

Educational strategies are needed to address the complexity of neuroanatomy and clinical neurology^21^ while minimizing cognitive load^22^ and enhancing comprehension and retention. Multimodal teaching methods have emerged as an approach to address these challenges. Multimodal education, defined as “*the instructional element presented in more than one sensory mode,*” includes the following learning environments: virtual, experiential, bedside, case-based, and team-based learning techniques.^23^ Multimodal learning facilitates the encoding, storage, and retrieval of new information^24-26^ and is grounded in cognitive memory theory.^27,28^ This learning strategy aims to encourage both knowledge acquisition and clinical skill development.^23,29,30^

Multimodal educational interventions in neurology can potentially improve neurology knowledge and reduce neurophobia.^31-33^ However, the impact of these educational strategies is not consistently examined. Few randomized controlled trials evaluate their efficacy and long-term impact.^34,35^ Most studies focus on short-term outcomes in knowledge or learner feedback, with limited exploration of how these interventions influence neurology knowledge, perception of neurology, and practical skills over time.

The aim of this study was to address this gap by evaluating neurophobia and neurology knowledge among final-year medical students before and after a multimodal neurology educational intervention called *Analogy, Switch to Clinical, Embody the Signs and Recall Learning* (*ANSWER*).

## Methods

### General Context

#### Study Design and Setting

**Figure 1:**
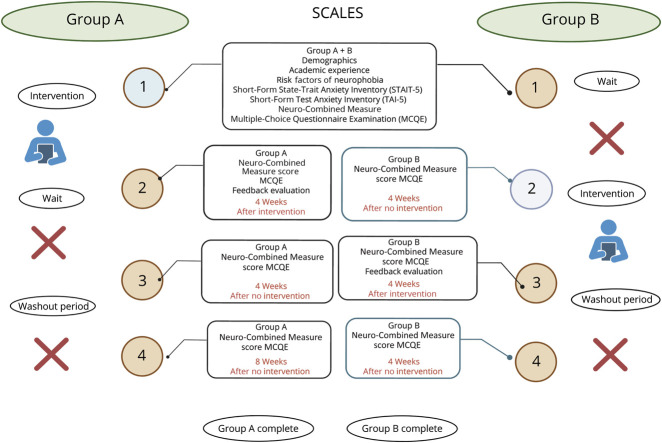
Study Design Flowchart The figure illustrates the randomized crossover design. Group A received the intervention first while Group B completed usual teaching during the wait period. Groups crossed over after 4 weeks. Assessments were conducted at 4 time points. At baseline (Time point 1), all students completed demographic measures, *STAIT-5, Test Anxiety Inventory (TAI-5), NCM*, and *MCQE*. The primary outcome (*MCQE*) and secondary outcome (*NCM*) were analyzed at Time point 2, comparing postintervention scores (Group A) with control scores (Group B). Time point 3 data were excluded because of dropout and crossover effects. A washout subgroup (n = 17) was reassessed at Time point 4 to evaluate retention and carryover effects. ANSWER = Analogy, Switch to Clinical, Embody the Signs and Recall learning; MCQE = multiple-choice question examination; NCM = Neuro-Combined Measure.

This study used a randomized crossover design to compare the *ANSWER* teaching intervention with usual teaching among final-year medical students at the Royal College of Surgeons in Ireland, Dublin, between September and December 2022. The study adhered to the CONSORT 2019 guidelines for randomized trials (Figure 1).^36^

*ANSWER* teaching was compared with usual teaching using a crossover design. This study design ensured that all students received equal teaching. Students were randomly assigned to groups A (intervention group: *ANSWER* teaching) and B (control group: usual teaching). An AB:BA design was used (in which the AB sequence is defined as intervention group followed by control group, and BA sequence is defined as control group followed by intervention group) and data were collected after each study period. Each study period comprised an eight-week teaching block: 4 weeks of *ANSWER* teaching and 4 weeks of usual teaching. There were 2 study periods. A carryover practice effect was considered.^37^ Group A's immediate postintervention assessments provided early insights into the intervention's effectiveness while Group B's initial waiting period ensured that any differences observed were attributable to the ANSWER intervention. Multiple outcome measures including neurophobia, knowledge acquisition, and program evaluation ensured comprehensive data collection while the washout period measured carryover effects. Participants were assessed at 4 time points: baseline (Time point 1), postintervention/control (Time point 2), post-crossover (Time point 3), and follow-up (Time point 4, washout subgroup only). Primary multiple-choice examination question (MCQE) and secondary Neuro-Combined Measure (NCM) outcomes were analyzed at Time point 2; Time point 3 data were excluded because of dropout and crossover effects while Time point 4 was used to assess retention in a subgroup of students.

### Standard Protocol Approvals, Registrations, and Participant Consents

Ethical approval was obtained from the local ethics committee (reference number: 202209004), and all participants provided written informed consent before enrollment.

### Study Design

#### Participant Recruitment and Randomization

**Figure 2:**
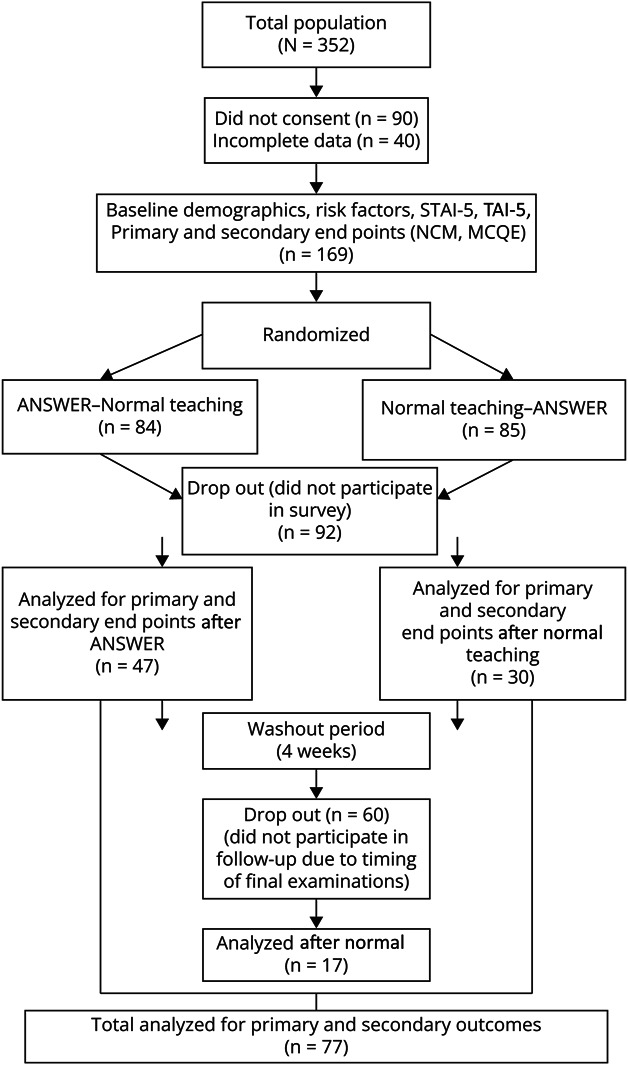
Consort Study Flow Of 352 eligible participants, 169 were randomized to either the ANSWER group (n = 84) or the normal teaching group (n = 85). After the intervention, 92 participants did not complete the survey and 60 were lost to follow-up because of final examinations. Primary and secondary outcomes were analyzed for 47 participants in the ANSWER group and 30 in the normal teaching group. After a 4-week washout period, 17 participants were analyzed. Outcomes included baseline demographics, risk factors, STAI-5 scores, Test Anxiety Inventory (TAI-5) scores, and academic performance measures (NCM, MCQE). Total analyzed for primary and secondary outcomes: n = 77. ANSWER = Analogy, Switch to Clinical, Embody the Signs and Recall Learning; MCQE = multiple-choice question examination; NCM = Neuro-Combined Measure.

The study invited final-year medical students to participate. Inclusion criteria comprised capacity to consent and fluency in English. Participants were randomly assigned to 2 groups using a 1:1 block randomization ratio, stratified by external staff not involved in the study. Randomization sequences were generated independently, and researchers were blinded to allocation. Data collection used pseudonymized identifiers to maintain participant confidentiality, as shown in Figure 2.

### Intervention and Control Descriptions

The control group received the usual teaching, comprising 2 clinical attachments and 2 medical teaching blocks. Teaching blocks were didactic lecture-based teaching sessions delivered by medical lecturers, covering all medical topics including neurology. Students attended 2 tutor-led bedside tutorials (maximum 5 students each), covering all medical topics including neurology.

The intervention group participated in the *ANSWER* teaching program, a multimodal educational program devised by the senior author (E.McG.). Two authors (E.McG. and L.McE.) created the teaching content. *ANSWER* stands for *Analogy*, *Switch to Clinical*, *Embody the Signs*, and *Recall the Learning*. Clinically relevant neuroanatomy was linked to the clinical encounter, enhancing student understanding of neurology and reducing neurophobia through this multimodal teaching method. The *Analogy* component used analogies to illustrate neuroanatomy. For example, when teaching students about seizures, the analogy of a lamp and a cable was used. To illustrate neuronal activity needed for normal cortical function, the electrical current from the cable to turn on the lamp was used. To illustrate abnormal electrical activity resulting in a seizure with loss of consciousness, a power surge down the cable leading to a power outage was used. The *Switch to Clinical* segment included clinical videos of neurologic signs, for example, a video EEG of a focal to bilateral tonic-clonic seizure. The *Embody the Signs* component required students to physically simulate neurologic signs, such as a focal motor seizure affecting the upper limb, promoting active learning through sensorimotor engagement. The *Recall* component involved MCQEs to reinforce the material, with weekly quizzes reviewing previous content. Each 90-minute session accommodated up to 88 students and covered 5 core topics: pyramidal syndrome, extrapyramidal syndrome, cerebellar syndrome, epilepsy, and peripheral neuropathy.

Mayer's multimedia learning principles, emphasizing cognitive load reduction through integration of verbal and visual information, were used to design the *ANSWER* intervention.^30,38^ These principles, including coherence, signaling, redundancy, and dual-channel processing, guided the creation of educational materials to optimize knowledge retention and skill acquisition. A consultant neurologist (EMG) and a neurology resident (LME) led the teaching sessions. Pre-reading was not required, and intervention content was accessible only during sessions. Different studies report curriculum development and feasibility. eTable 1 provides the *ANSWER* module structure.

### Outcome Measures

The primary outcome measures were student neurophobia and neurology knowledge. The secondary outcome measures were student feedback on the program.^39,40^

Neurophobia, defined as a “*fear of neurology and clinical neuroscience*,”^15^ arises from difficulty with the subject matter, limited student confidence, and low interest in neurology.^10,12,17,31,e1^ The *NCM* assessed neurophobia, a validated 9-item scale developed for the study. Through a comprehensive literature review and expert input from consultant neurologists, neuropsychologists, and academic staff, *NCM* items were developed. During item development, the published *Neuro-Q*^33^ and *Schon* questionnaires^e2^ were used. Each item is rated on a 5-point Likert scale, ranging from 1 = *strongly disagree* to 5 = *strongly agree*. Higher scores indicate a higher level of neurophobia. The *NCM* demonstrated strong internal consistency (Cronbach α = 0.80). Future studies will report psychometric data and NCM validation (eTable 2).

Neurology knowledge was measured using a 17-item single-best-answer *MCQE* designed by clinical neurologists and academic staff. Baseline knowledge in neuroanatomy, clinical historytaking, and interpretation of neurologic clinical signs were examined. Correct and incorrect responses were analyzed. Two pilot feasibility tests informed question refinement. Exclusion criteria included incomplete responses, ambiguous questions, and items with an average correct response rate exceeding 50%. The *MCQE* was not included in student formative assessments (eTable 3).

The Kirkpatrick model, a recognized framework for assessing teaching programs, evaluated the *ANSWER* teaching program. The Kirkpatrick model rates teaching programs against 4 levels of criteria: Level 1 (learner reaction), Level 2A (knowledge acquisition), Level 2B (skill performance), Level 3 (behavior change), and Level 4 (systemic impact and long-term learner outcomes). An anonymous digital survey after the intervention collected student feedback.^40,e3^ Responses were collected across 5 Likert scale categories (strongly agree to strongly disagree). Items were designed to align with Kirkpatrick levels; however, responses reflect self-reported perceptions and do not provide objective measures of behavior change or clinical impact.

**Table 1:** Participant Characteristics at Baseline and by Group Sequence

Variable	Total sample (n = 77)	Intervention group (n = 47)	Control group (n = 30)	Washout group (n = 17)
Age (y) Mean (SD)	25.45 (3.94)	26.17 (4.60)	24.40 (2.32)	24.94 (2.22)
Female, n (%)	50 (65)	28 (60)	21 (70)	12 (71)
Undergraduate entry, n (%)	49 (64)	26 (55)	20 (66)	12 (71)
Graduate entry, n (%)	28 (36)	21 (45)	10 (33)	5 (29)
NCM median (IQR) score, 95% CI	28 (6), (26.47–29.53)	29 (7), (26.96–31.04)	28.5 (8), (26.17–30.83)	28 (3), (26.01–29.99)
MCQE median (IQR) score, 95% CI	12 (4), (10.95–13.05)	11 (4), (9.84–12.16)	12 (5), (10.52–13.48)	13 (4), (11.07–14.93)
STAIT-5 median (IQR) score, 95% CI	13 (6), (12.01–13.99)	14 (7), (12.64–15.36)	13 (7), (11.19–14.81)	13 (4), (10.89–15.11)
TAI-5 median (IQR) score, 95% CI	13 (7), (11.59–14.41)	13 (7), (11.31–14.69)	13.5 (6), (12.00–16.74)	13 (6), (10.28–15.72)

Abbreviations: IQR = interquartile range; MCQE = multiple-choice questionnaire examination; NCM = Neuro-Combined Measure; STAIT-5 = State-Trait Anxiety Inventory Short-Form; TAI-5 = Test Anxiety Inventory Short-Form.

### Baseline Measures and Demographics

Baseline demographic data were collected using an online survey (SurveyMonkey) and included age, sex (male, female, nonbinary), and entry status (graduate or direct entry). In the Republic of Ireland, students can enter medical school through the undergraduate or graduate entry pathway. Undergraduate entry is available to students who complete secondary education and meet the necessary academic and standardized testing requirements. Students who have already completed a bachelor's degree in another field are eligible for graduate entry. Demographics, including self-identified sex, were collected from participants during data collection.

Baseline academic and trait anxiety were measured using 2 validated short-form instruments: the *State-Trait Anxiety Inventory* (STAIT-5)^34^ and the *Test Anxiety Inventory* (TAI-5).^35^ The STAIT-5 assesses stable, dispositional anxiety while the TAI-5 evaluates test-specific anxiety relevant to academic settings. Collecting measures of test and trait anxiety is relevant because both factors are reported to affect academic performance and cognitive functioning.^e4,e5^ These measures were included as covariates to examine whether individual differences in anxiety might influence neurophobia or interact with responses to the intervention. This decision was based on the hypothesis that test and trait anxiety may overlap with or contribute to neurophobia.

### Sample Size and Power Analysis

A priori power analysis was conducted using G*Power version 3.1.9.7 based on data^33^ from the validated Neuro-Q scale, assessing neurophobia, before and after a novel teaching method (n = 395). An effect size of *d* = 0.50 was selected, representing a medium effect per Cohen (1988) criteria.^e6,e7^ With α = 0.05 and power = 0.95 for a paired *t* test, the minimum required sample size was *N* = 54. A total of 352 students were invited to participate.

### Data Collection and Statistical Analysis

Data were collected at baseline and after each teaching period. Descriptive statistics, including median, interquartile range (IQR), mean, and SD, were calculated. Shapiro-Wilk tests assessed the distribution of all outcomes at baseline. Inferential statistics compare results between variables.

Given that the data distributions across groups were mixed, a nonparametric analysis was used for all groups to ensure reliable results. This approach aligns with statistical guidelines suggesting that nonparametric methods are appropriate when data do not meet the assumptions of normality, especially in mixed distribution scenarios.^e8^ To support the robustness of nonparametric summaries, bootstrapped 95% CIs were calculated for all medians using 1,000 resamples.

The Wilcoxon signed-rank test assessed within-group changes in neurology knowledge and neurophobia scores. Effect sizes were calculated using the rank-biserial correlation (*r*_ββ_), with values interpreted as negligible (0.00–0.09), small (0.10–0.29), medium (0.30–0.49), and large (0.50–1.00).

Baseline demographic and anxiety characteristics, including sex, were reported across groups following the CONSORT 2010 guidelines for randomized crossover trials.^e9,e10^ While randomization aims to balance characteristics, reporting and comparing baseline demographics such as age, sex, and academic background assess potential imbalances. Scale validation—including item development, content validity, confirmatory factor analysis, and Cronbach alpha—will be reported in future studies.^e11^

### Interim Analyses and Stopping Guidelines

Demographic distribution and baseline outcomes were analyzed before intervention commencement. Future studies will report observational curriculum feasibility findings from 2021 to 2024 that informed iterative intervention refinement. Stopping guidelines included participant-reported feedback rated as “poor” or a significant decline in *MCQE* scores.

### Data Availability

Anonymized data not published within this article will be made available by request from any qualified investigator.

## Results

### Descriptive Statistics

A total of 169 students were randomized, with 84 assigned to the ANSWER teaching group and 85 to the normal teaching group. Of these, 77 students completed all stages of the randomized crossover study. Final analysis excluded students with incomplete data.

Comparisons were made between students included in the final analysis (n = 77) and those excluded (n = 92). There were no significant differences between groups in gender distribution (χ^2^ (2, N = 169) = 3.53, *p* = 0.171) or student status (undergraduate vs graduate) (χ^2^ (1, N = 169) = 2.57, *p* = 0.109). However, a significant difference was observed in age (*z* = −3.20, *p* = 0.001), with included students being slightly older (median = 24 years, 95% CI [23.03–24.97]) than those excluded (median = 23 years, 95% CI [22.24–23.76]). No significant differences were found between groups in baseline scores for MCQE (*z* = −1.65, *p* = 0.098), NCM (*z* = −0.34, *p* = 0.737), STAIT-5 (*z* = −1.11, *p* = 0.266), or TAI-5 (*z* = 0.16, *p* = 0.876), with overlapping medians and CIs. Overall, included and excluded students were comparable across demographic and baseline outcome measures, except age.

Among the 77 students retained in the final sample, the mean age was 25 years (SD = 3.94, 95% CI [24.55–26.34]). Most were female (n = 50%, 65%) and entered through undergraduate admission (n = 49%, 64%); 28 students (36%) were enrolled through graduate entry (Table 2).

**Table 2:** Primary Outcome Measure Comparisons Before and After Teaching in Intervention, Control, and Washout Groups/Sequences

Outcome measure	Group sequence	Median (IQR) before	Median (IQR) after	z Value	*p* Value	Effect size (r_ββ_)
MCQE	Intervention	11 (3)	14 (4)	−4.82	<0.001	0.77
MCQE	Control	12 (5)	13 (5)	−1.78	0.075	0.3
MCQE	Washout	13 (4)	16 (1)	−3.51	<0.001	1.0
NCM	Intervention	29 (7)	26 (7)	2.87	0.004	0.51
NCM	Control	28.5 (8)	28.5 (6)	1.1	0.272	0.21
NCM	Washout	28 (3)	26 (4)	3.64	<0.001	1.0

Abbreviations: IQR = interquartile range; MCQE = multiple-choice question examination; NCM = Neuro-Combined Measure.

(*r*_ββ_) = rank-biserial correlation, a measure of effect size used in the nonparametric Wilcoxon signed-rank test. Effect sizes were categorized as negligible (0.00–0.09), small (0.10–0.29), medium (0.30–0.49), and large (0.50–1.00).

All 77 students completed baseline assessments for the NCM and MCQE. Median NCM scores at baseline were 29 (IQR = 7) in the intervention group, 28.5 (IQR = 8) in the control group, and 28 (IQR = 3) in the washout group. For MCQE, baseline median scores were 11 (IQR = 3) in the intervention group, 12 (IQR = 5) in the control group, and 13 (IQR = 4) in the washout group. One-way ANOVAs indicated no significant baseline differences between groups in TAI-5, STAIT-5, NCM, or MCQE scores (eTable 4).

### Neurophobia (NCM)

**Figure 3:**
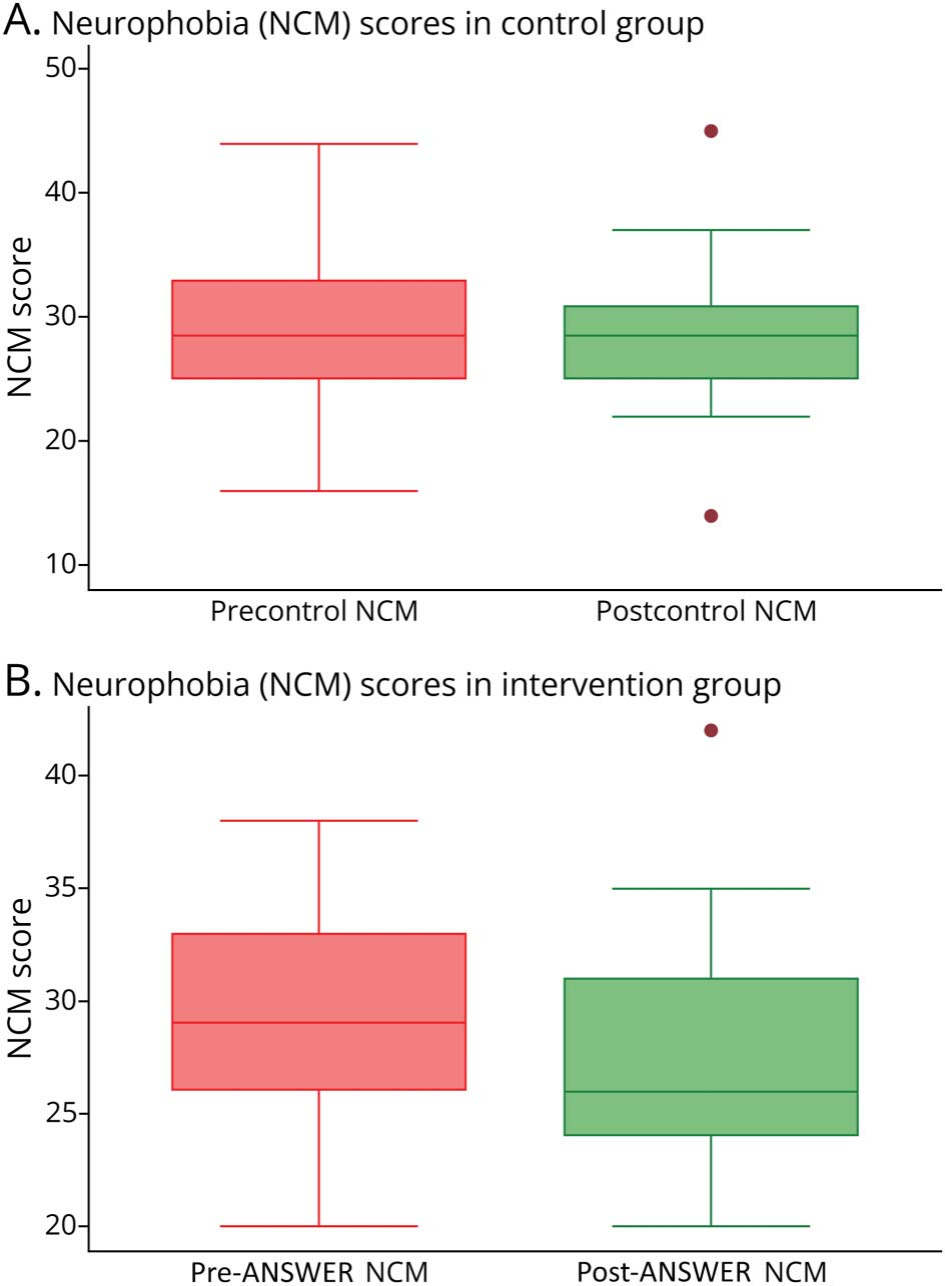
Neurophobia (NCM) Scores Before and After Teaching in Intervention and Control Groups Boxplot representations of the mean NCM score before and after control teaching (A) and ANSWER intervention (B). Neurophobia scores were significantly higher in the intervention group sequence and after the washout period. No significant difference was observed in the control group. (A) (Control group): The boxplot depicts the NCM scores before and after the control teaching. The median NCM scores appear similar before and after teaching, with no significant change observed. The interquartile range (IQR) remains relatively consistent, and the presence of outliers is noted. (B) (Intervention group): The intervention group's boxplot shows a reduction in NCM scores after the intervention. The median score is lower after teaching, indicating reduced neurophobia. The IQR is slightly narrower after the intervention, suggesting reduced variability in scores. Outliers are present, but the overall trend demonstrates a significant improvement. ANSWER = Analogy, Switch to Clinical, Embody the Signs and Recall Learning.

Neurophobia significantly decreased in the intervention group (n = 47), with median NCM scores declining from 29 (95% CI [26.94–31.06]) to 26 (95% CI [24.37–27.63]) (*z* = 2.87, *p* = 0.004, *r*_ββ_ = 0.51), indicating a large effect. This reduction was sustained in the washout group (n = 17), with NCM scores decreasing from a median of 29 (95% CI [27.96–31.33]) to 26 (95% CI [23.97–28.03]) (*z* = 3.64, *p* < 0.001, *r*_ββ_ = 1.00). By contrast, the control group (n = 30) showed no statistically significant change in neurophobia, with scores changing from a median of 28.90 (95% CI [26.73–31.07]) to 28.5 (95% CI [26.15–30.85]) (*z* = 1.10, *p* = 0.272, *r*_ββ_ = 0.21), with a small effect size (Figure 3 and Table 3).

**Table 3:** Kirkpatrick Model

Table for feedback, N = 129 (100%)							
	Question	Kirkpatrick^a^	Student response rate N (%)				
			Strongly agree (%)	Agree (%)	Neither agree nor disagree (%)	Disagree (%)	Strongly disagree (%)
1	“This teaching improved my neurological knowledge”	Level 2 (learning)	77 (60)	51 (39)	0 (0)	1 (1)	0 (0)
2	“The clinical videos are useful”	Level 1 (reaction)	82 (64)	44 (34)	3 (2)	0 (0)	0 (0)
3	“Lectures are engaging and easy to follow”	Level 1 (reaction)	79 (61)	41 (32)	8 (6)	1 (1)	0 (0)
4	“This teaching style will help me remember neurological concepts”	Level 2 (learning)	60 (47)	56 (43)	11 (9)	1 (1)	1 (1)
5	“I can apply this learning to my exams”	Level 3 (behavior^b^)	57 (44)	69 (53)	3 (2)	0 (0)	0 (0)
6	“I can apply this learning to my clinical skills”	Level 3 (behavior^b^)	46 (36)	81 (63)	2 (2)	0 (0)	0 (0)
7	“I can apply these clinical skills to a neurological patient”	Level 4 (results)^b^	44 (34)	80 (62)	5 (4)	0 (0)	0 (0)
8	“My learning was enhanced by the teaching style of educators”	Level 2 (learning)	46 (36)	72 (56)	9 (7)	2 (2)	0 (0)

Values represent number and percentage of students selecting each response (N = 129).

a The Kirkpatrick model was used as a guiding framework to structure question intent: Level 1 (reaction): learner engagement and satisfaction; Level 2 (learning): perceived knowledge gains; Level 3 (behavior): self-reported ability to apply learning; Level 4 (results): perceived ability to affect patient care.

b Items aligned with Levels 3 and 4 reflect self-reported perceptions and do not constitute direct measures of observed behavior or clinical outcomes.

### Neurology Knowledge (MCQE)

**Figure 4:**
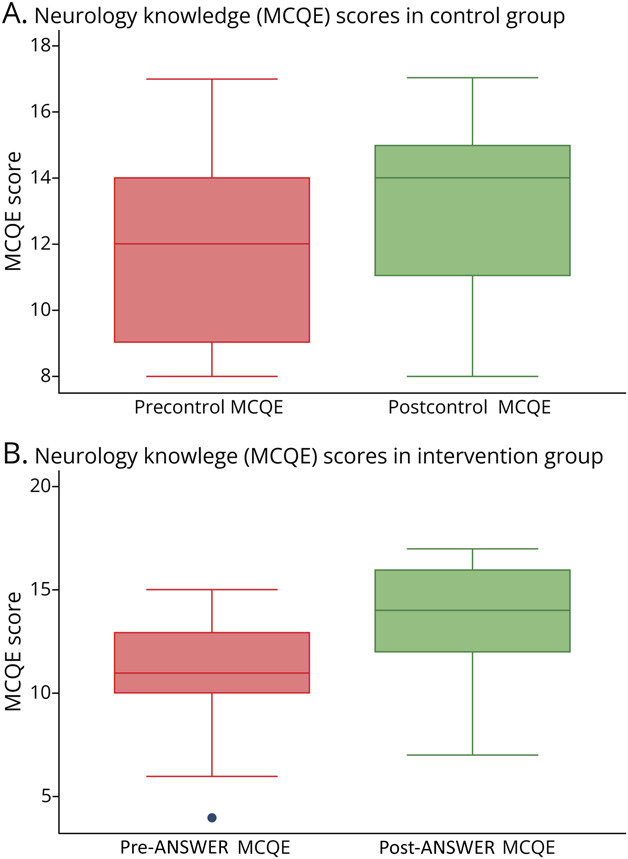
Neurology Knowledge (MCQE) Before and After Teaching in Intervention and Control Groups Boxplot representations of mean *MCQE* scores before and after control teaching (A) and ANSWER intervention (B). Knowledge acquisition scores were significantly higher in the intervention group. No significant difference was observed in the control group. (A) (Control group): The boxplot illustrates the *MCQE* scores before and after control teaching. Although there is a slight increase in the median score after teaching, the overall change is minimal and the interquartile ranges (IQRs) largely overlap, indicating no significant difference in knowledge acquisition. (B) (Intervention group): The intervention group shows improvement in *MCQE* scores after the ANSWER intervention. The median score is higher, and the IQR is narrower, reflecting increased consistency in performance. One pre-intervention outlier is observed, but the post-intervention scores demonstrate an upward trend. ANSWER = Analogy, Switch to Clinical, Embody the Signs and Recall Learning; MCQE = multiple-choice question examination.

The intervention group showed a significant improvement in neurology knowledge, with MCQE scores increasing from a median of 11 (95% CI [9.80–12.20]) to 14 (95% CI [12.57–15.43]) (*z* = −4.82, *p* < 0.001, *r*_ββ_ = 0.77). This improvement was maintained in the washout group (n = 17), where scores rose from a median of 13 (95% CI [11.09–14.91]) to 16 (95% CI [15.20–16.80]) (*z* = −3.51, *p* < 0.001, *r*_ββ_ = 1.00). The control group showed a nonsignificant change from a median of 11.43 (95% CI [10.17–12.69]) to 12.37 (95% CI [11.12–13.62]) (*z* = −1.78, *p* = 0.075, *r*_ββ_ = 0.30), indicating a small-to-medium effect (Figure 4 and Table 3).

### Program Evaluation

Seventy-six percent of the 169 randomized students (n = 129) completed the program evaluation. The overall mean rating for the teaching intervention was 35 of a maximum of 40, reflecting positive reception. Over 90% of students reported favorable responses, with clinical videos and analogy-based lectures rated as the most helpful components (98% and 93% agreement, respectively). Most students (94%) indicated that the teaching style improved their learning experience, and 90% reported enhanced memory retention. Table 1 presents a summary of student feedback.

## Discussion

The ANSWER teaching program uses multimodal teaching strategies to reduce neurophobia and improve neurology knowledge among final-year medical students. Consistent with previous studies on neurology education, which explored neurolocalization, clinical videography, experiential learning, simulation, and neuroanatomical models individually,^31,34, (e12–e20)^ the ANSWER method integrates these approaches into a comprehensive model to optimize student learning.

The information delivered used the theory of *germane load*^e21^ (*cognitive resources used to build schemas for long-term retention*) by creating a schema to enhance long-term memory. Scaffolding techniques (*beginning with foundational knowledge and progressively introducing complex topics*) are used. Active learning is present throughout the teaching process, and reflection is encouraged. Visual aids^e22^ mitigated complex topics into manageable units through structured frameworks.^e23^ Theoretical models,^e24,e25^ such as deep approach to learning,^e26,e27^ encourage motivation to learn and engage with neurologic concepts. Clinical video demonstrations bridged theoretical knowledge with clinical application,^e28,e29^ facilitating the recognition of neurologic signs. Embodying neurologic signs—actively imitating physical manifestations—can minimize cognitive load^e30^ and support the encoding and recall of acquired knowledge^e30-e32^. Retrieval-based learning enhances retention.^e33^ Spaced repetition^e33^ and adaptive learning platforms ensured that students reinforce key concepts over time.^e34,e35^

We used a randomized crossover design to examine the effects of the ANSWER teaching program on neurophobia and neurology knowledge. Randomized crossover trials are underused in neurology medical education research.^34,e36^ Crossover design allows students to serve as their own control, reducing variability and enhancing internal validity.^36^ In addition, it provides evidence without limiting access to educational interventions, making it well suited for curriculum studies.^e37^ Repeated assessments may increase dropout rates and incomplete data.^e38^

We used neurophobia and neurology knowledge as our primary study outcomes. An evaluation incorporating both academic and psychological measures may provide a more accurate assessment of curricular efficacy.^e39^ Standardized, repeatable end points improve comparability across studies and advance the field of neurology education. Consistency in outcome measures remains challenging,^34^ underscoring the need for robust, long-term evaluations of educational interventions with validated instruments. While the study provides evidence for the effectiveness of the *ANSWER* intervention, it also highlights the need for further research into end points in neurology education. Current literature often lacks consistency in outcome measures, making comparing studies and drawing definitive conclusions challenging. Future research will explore the relationship between academic anxiety, trait anxiety, and validated neurophobia scales in medical students.

Our study should acknowledge several limitations. Logistical time frames associated with formative assessments and the increased workload of final-year medical students constrained the sample size. The dropout rate was attributed to students approaching formative assessments, limiting their availability to complete repeatable measures. In addition, the study was conducted within a single institution, potentially limiting the generalizability of findings to other medical schools or health care settings. While the program evaluation followed the Kirkpatrick model, Levels 3 (behavior) and 4 (results) relied on self-reported perceptions rather than objective measures of behavior or patient care measures. Furthermore, the research design did not include longitudinal follow-up beyond the study period.

This study adds to the evolving field of neurology education by demonstrating how multimodal, student-centered teaching can improve knowledge and foster confidence and positive attitudes toward neurology. Future research should focus on replicating these findings across multiple institutions and exploring their impact on long-term clinical competency and career trajectories. Our findings highlight the promise of innovative educational approaches such as *ANSWER* in neurology education.

## Glossary

ANSWER: Analogy, Switch to Clinical, Embody the Signs and Recall Learning
IQR: interquartile range
MCQE: multiple-choice examination question
NCM: Neuro-Combined Measure
STAIT-5: State-Trait Anxiety Inventory
